# The nature of label-induced categories: preverbal infants represent surface features and category symbols

**DOI:** 10.1098/rspb.2024.1433

**Published:** 2024-11-20

**Authors:** Barbara Pomiechowska, Szilvia Takács, Ágnes Volein, Eugenio Parise

**Affiliations:** ^1^ Centre for Developmental Science & Centre for Human Brain Health, School of Psychology, University of Birmingham, Birmingham, UK; ^2^ Cognitive Development Center, Department of Cognitive Science, Central European University, Quellenstrasse 51, Vienna 1100, Austria; ^3^ CIMeC - Center for Mind/Brain Sciences, University of Trento, Trento, Italy; ^4^ Department of Psychology, Lancaster University, Bailrigg, Lancaster LA1 4YF, UK

**Keywords:** infants, categorization, labelling, EEG, alpha-band ERD

## Abstract

Humans categorize objects not only based on perceptual features (e.g. red, rounded), but also function (e.g. used to transport people). Category membership can be communicated via labelling (e.g. ‘apple’, ‘vehicle’). While it is well established that even preverbal infants rely on labels to learn categories, it remains unclear what is the nature of those categories: whether they simply contain sets of visual features diagnostic of category membership, or whether they additionally contain abstract category markers or symbols (e.g. linguistic in the form of category labels or non-linguistic). To address this question, we first used labelling to teach two novel object categories, each composed of unfamiliar visually unrelated objects, to adults and nine-month-olds. Then, we assessed categorization in an electroencephalography category-oddball task. Both adults and infants displayed stronger neural responses to the infrequent category, which, in the absence of visual features shared by all category members, indicates that the categories they set up contained feature-independent category markers. Well before language production starts, labels help infants to discover categories without relying on perceptual similarities across objects and build category representations with summary elements that may be critical for the development of abstract thought.

## 1. Introduction

Humans learn uncountable artefact categories. Categorization of artefacts depends not only on their physical characteristics but also on their function. Similar-looking objects often fall into discrete non-overlapping categories, underpinned by different artefact concepts. For example, knives, wrenches, toothbrushes and pens have a similar shape and length, but they are typically categorized as instances of distinct object kinds, each characterized by a different function and lexicalized separately under a different name. Therefore, mastering a broad range of artefact concepts and categories available within one’s cultural community requires going beyond perceptual features of the stimuli and constitutes a major learning challenge.

One powerful strategy to discover relevant artefact concepts and categories is supplied by language. Language offers special devices, in the form of category labels, that directly inform language users how to categorize non-linguistic experience (for reviews [1–3]). Labelling indicates which entities are members of the same category (i.e. those that receive the same name, e.g. ‘dog’, ‘moxi’) and which ones belong to a different category (i.e. those that receive a different name, e.g. ‘cat’, ‘wug’). Moreover, by attending to labels learners can discover culturally relevant groupings of objects, even before they appreciate the underlying concepts (e.g. what ‘dogs’ or ‘moxis’ are).

The earliest use of labels as category markers has been documented as early as 3 to 4 months of age [4,5] in very young infants who have only just begun to acquire language. Infants familiarized with images of eight different exemplars of one category (e.g. ‘dinosaur’), each presented in conjunction with a label (e.g. ‘Look at the toma! Do you see the toma?’), displayed evidence of categorization in a preferential-looking test contrasting a new member of the familiarized category (i.e. a previously unseen dinosaur) with a member of a different category (e.g. a fish) by looking longer at the test image representing the novel category. As children grow older and become more syntax savvy, they develop expectations that different parts of speech mark different kinds of categorical distinctions, linking nouns specifically to object kinds and adjectives to object properties [6–8]. People continue to rely on labels to discover new categories throughout their lifespan (e.g. [9–13]).

Despite the importance of labelling for category learning, to date, neither the cognitive mechanisms that support label-guided categorization early in life, nor their neural markers, have been fully understood. Previous infant studies have focused on identifying what aspects of object processing may be shaped by the presence of a label. Four main findings emerge from this literature. First, facilitatory effects on categorization seem not to be specific to labels *per se*, but can also be elicited by other auditory stimuli that infants perceive as communicative. In the first four months of life, these include non-human primate calls [5]. Later on, infants succeed to use non-linguistic sounds (e.g. beeps [14]) to discover new categories when they are given evidence that those sounds are communicative (e.g. after being exposed to an adult replying to another through beeps). Second, labelling influences the online distribution of infants’ visual attention. When multiple objects receive the same name, infants focus on commonalities among them [15], which helps them to divide continuous visual input into discrete categories, whether presented with visual stimuli on a morphed continuum [16,17] or with correlated feature structures [18]. The presence of labels can also override spontaneous visual categorization and impose new category boundaries (see experiments 4–5 in [18]). This may be owing to the fact that labelling exerts a top-down supervisory influence on visual processing (cf. [19], cf. [20, 21–23]). Third, labelling affects what object features are encoded in the working memory. Convergent behavioural and electrophysiological findings suggest that when the same label accompanies different category tokens, infants preferentially encode the shared features characterizing the presented object set, thus leading to category formation [24]. Conversely, when distinct labels accompany each token, infants prioritize encoding individual-specific features, thus enhancing object individuation [24,25]. Finally, during the second year of life, labelling takes priority over featural information when children determine category boundaries, supporting inferences about function [26,27], numerosity [28] and reference [29].

Critically, however, it is unknown what is the nature of categories that infants set up in response to labelling. The literature indicates that they form feature-based representations [4,9,15,16,30,31], but leaves open whether these representations involve other components such as labels themselves, or non-linguistic category markers, or symbols that act as category placeholders. In adults, category representations include labels that people spontaneously access even when passively viewing images of objects [19,32,33], and non-linguistic indexes [34] that become activated by category-diagnostic content, provide access to the long-term semantic knowledge, and can be thought of as non-linguistic symbols evidenced to support higher-order compositional thinking outside of natural language [35]. An evolutionary precursor of such representational structure may be present in non-human primates, as indicated by strong categorical responses unaffected by featural differences between individual category tokens observed in the prefrontal cortex of rhesus monkeys [36,37]. There is initial developmental evidence that infants can set up non-linguistic category symbols that support object encoding as well as working memory storage [38], and seem to be able to use rudimentary linguistic symbols as early as between 6 to 9 months of age [39–42]. Note also that infants show evidence of word extension for common nouns (e.g. [43,44]), but not for proper names (e.g. [44]), a process which in adults is probably supported by having category labels integrated into category representations. However, it remains unclear what is the mechanism of infant word extension. It is possible that infants only store label-object (and not label-category) associations, and succeed to generalize words to previously unseen objects by first retrieving the visual representation of the object associated with the familiar word (e.g. the representation of their own hand or their carer’s hand), and then identifying the most similar item on the display.

Here, our goal was to experimentally assess what elements infants include in their category representations and deploy when visually exploring their environment. More specifically, we asked whether in addition to extracting and storing the relevant visual features of the observed category tokens, infants recruit rudimentary symbolic elements, which could take the form of either category labels or non-linguistic category indexes. To this aim, we taught nine-month-olds two categories, each consisting of novel objects that did not share visual features, and then tested their categorization performance using a novel electroencephalography (EEG) category oddball task. Below, we introduce, in turn, the motivation behind each of these methodological choices.

Past studies examining the effects of labelling on category formation during the first year of life have only used objects that were visually similar (i.e. sharing features such as shape or texture), either drawn from real-life categories (e.g. car, airplane, cow, dinosaur, animal, vehicle and tool [10]), dinosaur and fish [14], animal, fruit and vehicle [45], or artificially created ones (e.g [16,17,18]). Such stimuli make it very difficult, if not impossible (cf. [36], tasks using single-cell recordings [37]), to probe the content of underlying category representations. This is because featural contents co-vary with potentially co-existing higher-level summary signals, thus leaving it ambiguous whether the observed categorical responses are owing to processes operating on the featural or supra-featural level. One way to probe the presence of abstract content in category representations is to use categories made of objects that jointly do not share any common visual features. Any categorical responses observed in such context could only be triggered by supra-featural elements of category representations, such as labels or non-linguistic symbols, activated by seeing objects that belong to category tokens. Guided by these considerations, we used novel categories composed of unfamiliar objects carefully selected to have no overlap in visual features. That young infants may set up categorical representations that cut across visual similarities and/or are agnostic to visual similarities between objects gains plausibility from the findings that early during the second year of life, children rely on labels rather than visual features to make inferences about function [26,27] and numerosity [28].

Traditional behavioural methods for studying infant categorization do not allow for assessing categorization of visually unrelated objects. This is because these methods rely on habituation (or familiarization) procedures followed by generalization tests probing discrimination between a previously unseen category token and a token of a different category (e.g. [4,5]). The visual similarity between the category token used at test and the training set is a prerequisite of generalization, thus, such tests cannot be used for testing arbitrary visual categories. To address this limitation, we used an electrophysiological event-related *category-oddball* task ([19,32,46,47], but see also [48–50], for frequency-tagging category-oddball tasks). Oddball paradigms are typically employed to measure frequency-dependent responses in scalp-recorded EEG [51–53] through stimuli presentation that involves interleaving frequent stimuli (e.g. 75%) with infrequent stimuli (e.g. 25%). The detection of infrequent stimuli is indexed by event-related responses, event-related potentials (ERPs)(e.g. higher amplitude P300, [53]), or event-related induced oscillatory activity (e.g. stronger desynchronization in the alpha frequency band to infrequent relative to frequent stimuli [47,54]).

In our *category-oddball* task, participants saw images of objects from two newly learned categories: a frequent one (i.e. category 1 displayed 75% of time, comprising *object1, object2, object3*) and an infrequent one (i.e. category 2 displayed 25% of time, comprising *object4*). The asymmetry in the frequency of presentation was at the category level, while all four individual objects were displayed equally often (i.e. 25% of time). Therefore, frequency-dependent oddball responses could arise only if the stimuli were mentally sorted into two distinct categories, and, as such, provide evidence of categorization. Applying this task to categories composed of featural unrelated objects ensured that category oddball responses cannot result from processing perceptual features. Instead, the only shared property of individual objects is a category-level symbol indicating their category membership. Thus, the presence of oddball responses for arbitrary categories would provide evidence for the deployment of category symbols in the infant brain, or, in other words, for the supra-featural components of category representations. To assess oddball responses, we derived two EEG measures, selected based on the literature: the P300 ERP component and the total-induced oscillatory activity in the alpha-band. We expected the infrequent stimuli to elicit a higher P300 amplitude [52,53] and/or a stronger alpha-band event-related desynchronization (ERD) [47,52–54].

We conducted three EEG category-oddball experiments using different stimuli sets and different age groups. We first validated the oddball task in adults (experiments 1–2), and then tested infants (experiment 3). In experiment 1a (and its direct replication, experiment 1b) we used familiar categories. In experiment 2, we used labelling to teach adults two novel arbitrary categories composed of unrelated and unfamiliar objects prior to administering the EEG category-oddball task. In experiment 3a (and its direct replication, experiment 3b), we used the same stimuli and category-oddball task as in experiment 2, but modified the category training, replacing a computerized task with an infant-friendly live training session.

## 2. Experiment 1: neural markers of categorization

The aim of experiment 1 was to validate the EEG category-oddball task. We presented adults with images of four familiar objects: a spoon, a fork, a knife and a hammer, while recording their EEG in two independent experiments: experiment 1a, and its direct replication, experiment 1b. To test whether participants spontaneously grouped the spoon, the fork and the knife into a single category (i.e. cutlery) that included these objects but not the hammer, we computed ERPs and ERD to the frequent (cutlery) and infrequent (hammer) categories.

### (a) Methods

#### (i) Participants

Thirty adult paid volunteers (mean age = 23.8 years, range = 19–37 years, 16 females) participated in this research (experiment 1a: *n = 15;* experiment 1b: *n* = 15). The sample size was established on the basis of the large effects that oddball paradigms produce on the P300 [53]. The procedure of all experiments was approved by the Hungarian United Ethical Review Committee for Research in Psychology EPKEB. Before the EEG session, participants were informed about the procedure and gave written consent. All the experiments were conducted according to the principles of the Declaration of Helsinki.

#### (ii) Stimuli

**Figure 1.**
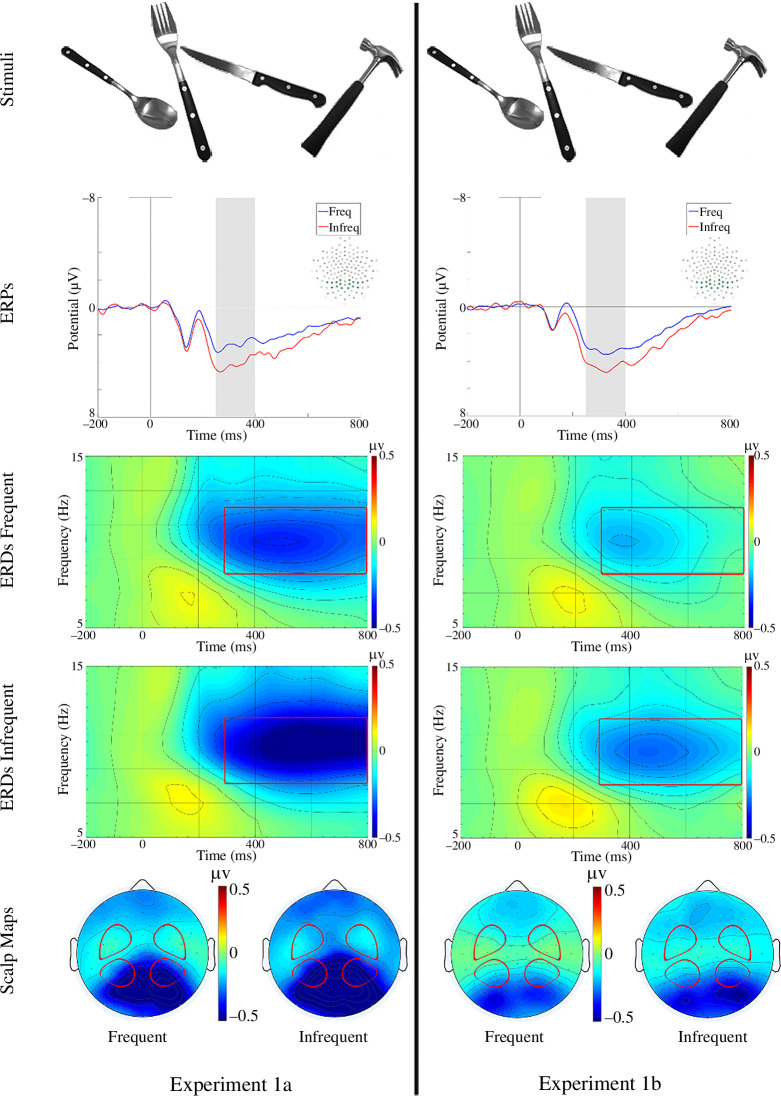
Procedure and results of experiment 1. Stimuli panels provide example images of all four objects used in the experiment. ERPs illustrate the average waveforms across all analysed channels. The grey barrels indicate the time window for the P300 component. ERDs display the average desynchronization across all channels analysed (see scalp maps panels below). The red rectangles highlight the time-frequency window used in the analyses. Scalp maps panels display the average activity in the given time-frequency window used in the analysis; each condition is pictured separately. Red shapes indicate the four regions of interest used in the statistical analyses.

The stimuli were images of four objects: a fork, a knife, a spoon and a hammer (figure 1, top). Each object was photographed from four different angles for a total of 16 images. The objects were presented on grey background and the images were edited to approximately equate object sizes and luminance levels.

#### (iii) Procedure

Images were presented in the centre of a computer screen. Each individual object appeared with the same probability (25%) in a pseudo-random order. The transitional probability between any two objects was 33.3% (i.e. no two consecutive trials presented the same object). The four different views of each object varied randomly across trials. The stimuli were presented for 800 ms. Each test image was immediately preceded by a fixation stimulus (for a total of 1040 to 1240 ms: first rotating for 640 ms and then still for 400 to 600 ms, randomly determined). Six different animations were used as fixation stimuli, changing every 16 trials. Between trials, the screen remained blank (800 to 1000 ms, randomly determined).

Participants received three blocks of 100 trials each, with short pauses in between blocks. They received instructions to pay attention to the stimuli, but were not requested to respond to them in any way.

#### (iv) Electroencephalography recording and analyses

High-density EEG was recorded continuously using Hydrocel Geodesic Sensor Nets (Electrical Geodesics Inc., Eugene, OR, USA), including 128 electrodes referenced to the vertex (Cz). Signals were acquired using an Electrical Geodesic Inc. amplifier GES 300 at a sampling rate of 500 Hz with a low-pass filter at 200 Hz. The EEG was offline band-pass filtered between 0.3 and 30 Hz and segmented into epochs including 400 ms recording before and 1000 ms after stimulus onset^1^ . Epochs were automatically rejected whenever the average amplitude of an 80 ms sliding window exceeded 55 μV at horizontal electrooculogram (EOG) channels, 140 μV at vertical EOG channels (only the recording with adults included this information) and 200 μV at any other channel. Bad channels were automatically interpolated in epochs in which 10% or less of the channels contained artefacts; epochs in which more than 10% of the channels contained artefacts were automatically rejected.

To measure oscillatory EEG responses, the artefact-free segments were subjected to time-frequency analysis in MATLAB^®^. The epochs were re-referenced to average reference and were convoluted by complex Morlet wavelets for the frequencies 5–15 Hz with 1 Hz resolution using a custom-made script collection, WTools (available upon request). The absolute values of the complex coefficients were computed, and the epochs were baseline-corrected to the 200 ms immediately preceding stimulus onset.

Based on the literature [53], as well as on visual inspection, we quantified the amplitude of the P300 response as the average ERPs within two parietal regions of interest (ROIs) between 250 and 400 ms after the stimulus onset. We did not have enough prior evidence to develop specific predictions about topology of the hypothesized category-related alpha ERD effects, but we predicted a stronger alpha-band ERD for the oddball compared to the standard category. We measured the alpha-band ERD at four ROIs corresponding to the anterior and posterior parts of the two hemispheres as the average amplitude in the time window between 300 to 800 ms after the stimulus onset in the frequency range of 8–12 Hz. Further details of EEG editing and channel selection are reported in the electronic supplementary material.

### (b) Results

In experiment 1a, the images of hammers belonging to the infrequent category elicited a P300 with larger amplitude than the images of other stimuli belonging to the frequent category (figure 1, left column). An ANOVA on the P300 amplitude with category (frequent versus infrequent) and hemisphere (left versus right) as within-subjects factors revealed a main effect of category (*F*_1,14_ = 34.79, *p* < 0.001, $\eta_{p}^{2}$ *=* 0.71), with the infrequent stimuli eliciting a more positive P300. There was no main effect of hemisphere, *p* = 0.149, but we found a marginal interaction category x hemisphere; see the eletronic supplementary material for details. In addition, the images from the infrequent category elicited a pronounced ERD in the alpha band. The ERD was analysed with category (frequent versus infrequent), hemisphere (left versus right) and region (anterior versus posterior) as within-subjects factors. We found a main effect of category: the images of the infrequent category objects produced a stronger attenuation of alpha oscillations than those from frequent category, *F*_1,14_ = 9.09, *p* = 0.009, $\eta_{p}^{2}$ *=* 0.39; for further details see the electronic supplementary material. We also found a main effect of region, *F*_1,14_ = 10.58, *p* = 0.006, $\eta_{p}^{2}$ *=* 0.43, suggesting stronger alpha attenuation over the posterior than anterior regions. We found no other main effect or interaction, all *p*s > 0.09.

Experiment 1b (figure 1, right column) was a direct replication of experiment 1a, using the very same procedure and analysis pipeline. We observed significant effects of category on both the amplitude of the P300, *F*_1,14_ = 32.46, *p* < 0.001, $\eta_{p}^{2}$ *=* 0.70, and the attenuation of alpha-band oscillations, *F*_1,14_ = 8.65, *p* = 0.011, $\eta_{p}^{2}$ *=* 0.38. In the P300 analysis, there was no main effect of hemisphere, *p* = 0.151, but there was an interaction category x hemisphere (for further details please see the electronic supplementary material). As in experiment 1a, the ERD analysis also yielded a main effect of region, *F*_1,14_ = 6.13, *p* = 0.027, $\eta_{p}^{2}$ *=* 0.30. We found no other main effect or interaction, all *p*s > 0.13.

We directly compared P300 and alpha-band ERD between the two experiments (1a−1b) with the additional between-subjects factor of experiment (1a versus 1b). We found an effect of category, *F*_1,28_ = 67.08, *p* < 0.001, $\eta_{p}^{2}$ *=* 0.71, a main effect of hemisphere, *F*_1,28_ = 4.61, *p* < 0.042, $\eta_{p}^{2}$ *=* 0.14, and a category x hemisphere interaction, *F*_1,28_ = 8.96, *p* = 0.006, $\eta_{p}^{2}$ *=* 0.24, on the amplitude of the P300. There was no effect of, or interactions with, experiment or any other significant effect or interaction, all *p*s > 0.46. As for the ERD, we found a main effect of category, *F*_1,28_ = 16.82, *p* < 0.001, $\eta_{p}^{2}$ *=* 0.38, a main effect of region, *F*_1,28_ = 15.38, *p* < 0.001, $\eta_{p}^{2}$ *=* 0.36, and an interaction of experiment x region, *F*_1,28_ = 4.64, *p* = 0.04, $\eta_{p}^{2}$ *=* 0.14. There was no other effect or interaction, all *p*s > 0.082.

### (c) Discussion

The stronger neural responses to stimuli from the infrequent category suggest spontaneous categorization of the depicted objects into two categories: a frequent one (cutlery) and an infrequent one (hammer). However, it remains unclear whether these frequency effects were owing to the perceptual features of our stimuli that made the hammer stand out from the other items or to the activation of category symbols. To disambiguate what drives the observed effects, we ran experiment 2 (but see also experiment S4 in the electronic supplementary material). We trained adults to group together objects that shared no common visual features, but belonged to two label-based, arbitrary and artificial categories. Then, we tested them with the category-oddball EEG test.

## 3. Experiment 2: learning novel categories through labelling

Experiment 2 tested whether the EEG frequency effects observed in experiment 1 were owing to categorization based on non-featural symbolic content, such as labels or non-linguistic category placeholders, or on sets of visual features diagnostic of category membership. We taught participants two arbitrary categories, each consisting of three unrelated objects. Each category was introduced by a distinct label in the form of a pseudoword unfamiliar to the participants. Subsequently, we measured their brain activity during a category-oddball task using four out of the six objects, three of them previously introduced with one label (frequent category), and one with the contrasting label (infrequent oddball category).

### (a) Methods

#### (i) Participants

Fifteen adult paid volunteers (mean age = 22.5 years, age range = 19–30 years, nine females) participated in this experiment. We maintained the same sample size as in experiment 1. Participants were monolingual Hungarian speakers.

#### (ii) Stimuli

**Figure 2.**
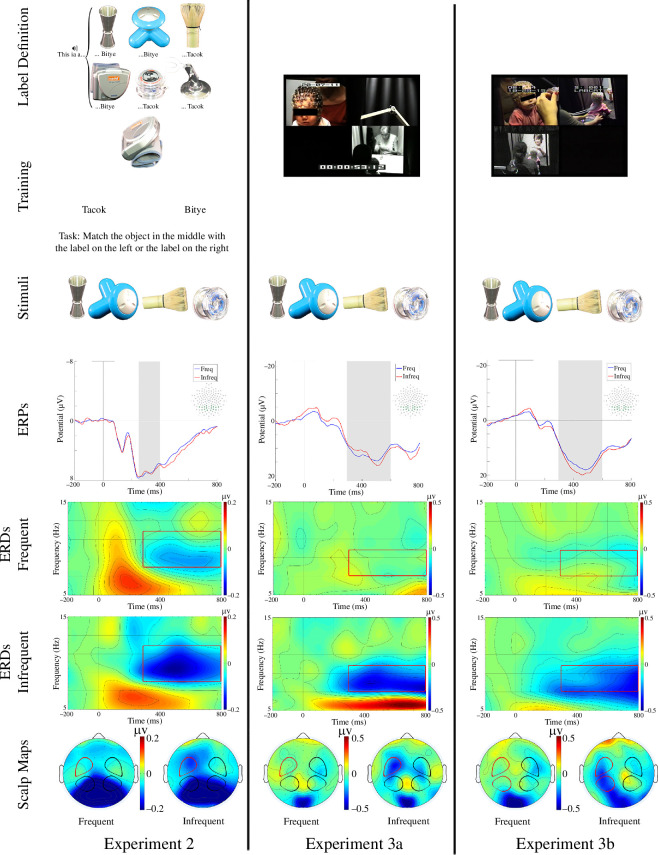
Procedure and results of experiments 2 and 3. Label definition panels display an example of the label-object pairings. Adults (experiment 2) received these pairings on a computer screen; infants (experiments 3a-b) received live training. Training panel provides an example of training trials (experiment 2). Stimuli panels provide examples of the images displayed during the EEG recording. ERP panels illustrate the average waveforms across all analysed channels. The grey barrels indicate the time windows analysed for the P300 component. ERD panels display the average desynchronization across all channels in the left ROI (see scalp maps panels below). The red rectangles highlight the time-frequency windows used in the analyses. Scalp maps panels display the average activity in the given time-frequency window for the two conditions separately. Shapes on the maps indicate the location of four ROIs used in the statistical analyses. The red shape indicates the ROI of the ERD effects displayed.

Six artefacts were employed in the training phase (see figure 2). Each was photographed from four different orientations. The resulting images were edited in the same way as in experiment 1, and were used as stimuli in both the training and in the test phase. Two phonotactically valid Hungarian pseudo-words were used in the training phase: *tacok* and *bitye*.

The objects were grouped into two categories of three objects each. The grouping was counterbalanced across participants. Therefore, although some pairs of individual items may have shared common visual features (e.g. metallic surface), there were no obvious unifying features at the category level, i.e. characterizing trios of objects.

#### (iii) Procedure

Experiment 2 consisted of two phases: category training and EEG category-oddball task. The participants were prepared for the EEG recording before the training and proceeded to the oddball task immediately after completing the training phase.

##### (i) Category training

The category training had two parts, label exposure followed by a label test. The entire training was repeated until participants learned both novel categories. In the label exposure phase, participants were presented with single individual images of each of the six objects (duration: 1 s; order randomized). Each image was labelled by a female voice as either tacok or bitye (e.g. ‘this is the *tacok*’; Hungarian: ‘ez a *tacok*’, figure 2, top left). Each participant received a different label-defined grouping of the six objects. In each trial of the label test, one of the six objects was presented in the middle of the screen, and the words tacok and bitye were displayed in the lower left and right corners (counterbalanced). Participants were instructed to categorize the object, by pressing one of two buttons on a gamepad. Responses were followed by visual feedback highlighting the correct label. After 12 trials, in which participants had to decide about each of the six objects twice, they were informed about the number of committed errors. If they made any errors, the entire category-training phase was repeated. The category-training phase ended when the participant demonstrated learning of both novel categories, answering correctly on 12 consecutive trials. On average, participants took 3.6 category training rounds (s.d. = 1.76) before proceeding to the EEG category-oddball task.

##### (ii) Electroencephalography category-oddball task

During the EEG category-oddball task, participants were presented with images of four objects: a set of three objects sharing one label (frequent category) and a randomly chosen object named with the other label (infrequent category). As in experiment 1 objects images appeared in a pseudo-random order, each individual object appearing with a 25% probability, with a transitional probability of 33.3% between any two objects. The four different views of each object varied randomly across trials. The two categories (i.e. the one corresponding to the word *tacok* and the one corresponding to the word *bitye)* were equally likely to be the frequent category at test.

Trial duration, inter-trial interval, number of trials per block, number of blocks, EEG recording parameters, editing and analysis methods were the same as in experiment 1.

### (b) Results

We used the same analyses as in experiment 1 and found no difference between conditions in the P300 response (all *p*s > 0.17; figure 2, left column). The analysis of the alpha-band ERD, however, revealed two significant interactions: between category x region, *F*_1,14_ = 12.11, *p* = 0.004, $\eta_{p}^{2}$ *=* 0.46; category x hemisphere x region, *F*_1,14_ = 4.70, *p* = 0.048, $\eta_{p}^{2}$ *=* 0.25. We did not find any other significant effects, all *p*s > 0.068. To resolve the interactions, we ran four separate paired-sample *t*-tests within each region of each hemisphere, comparing the ERD activation elicited by the presentation of the frequent and infrequent categories. The objects from infrequent category elicited stronger alpha desynchronization compared to the object from the frequent category, *t*_14_ = 5.08, *p* < 0.001, *d* = 0.48, only in the anterior left area; other regions, *p*s > 0.6 (figure 2, left column).

### (c) Discussion

This pattern of results warrants two observations. First, the absence of a P300 effect suggests that this ERP component is more sensitive to frequency differences at the level of perceptual features than categorical representations. Second, the presence of the ERD effect confirms that this neural marker reflects categorization and does not depend on the physical characteristics of the stimuli. Because categories in this task were arbitrary groupings of visually unrelated objects, each arguably eliciting a different pattern of visual activity, the observed frequency response must have been mediated by a higher-level representation common across different objects such as a category label or a non-linguistic category symbol. The categorization effect on the ERD was largely restricted to the left anterior areas of the scalp, while it was more pronounced over the posterior regions in experiment 1. This shift in topology of the effect could be owing either to the difference in familiarity (familiar categories in experiment 1 versus novel categories in experiment 2) or to the presence of the training phase (i.e. there was no training phase in experiment 1) that included explicit labelling of unfamiliar objects.

## 4. Experiment 3: learning novel categories through labelling in infancy

To investigate the nature of categories infants set up in response to labelling, we tested nine-month-olds in two experiments, 3a, and its direct replication, 3b. We used the same visual stimuli and categorization test as in experiment 2, but we modified the category-training phase to make it infant-friendly. We used a live training, during which an experimenter showed infants the real three-dimensional objects, whose photos were used in experiment 2. She presented each object individually for 1 min, while labelling it to induce category formation. Three objects were named by one pseudoword, and the three other objects by another one. Informed by past evidence, we reasoned that infants will categorize together objects that receive the same name. Following category training, infants were administered the EEG category-oddball test, to assess their categorization performance.

### (a) Methods

#### (i) Participants

Fourteen, nine-month-olds (mean = 278 days, range = 267–288 days, three females) participated in experiment 3a and 20 nine-month-olds (mean = 271 days, range = 256–282 days, 11 females) participated in experiment 3b. All infants came from monolingual Hungarian-speaking families. Thirty additional infants were excluded because of fussiness and crying (*n* = 12), insufficient number of artefact-free EEG trials (i.e. less than nine, *n* = 13), excessive movement artefacts (*n* = 1), not matching the selection criteria (recognized as born preterm after testing, *n* = 1), experimenter’s error (*n* = 2), technical problems (*n* = 1). Such an attrition rate is typical for infant EEG studies [55]. The sample size for experiment 3a was determined *a priori* on the basis of earlier studies with infants of similar age [39]. For the replication in experiment 3b, we increased the sample size to elevate the power of the experiment. Parents were informed about the procedure and gave written consent before the experimental session.

#### (ii) Stimuli

Six artefacts were employed in the training phase (see figure 2). All of them were novel to infants. The images of these objects, already used as stimuli for adults in experiment 2, were used in the test phase. The two pseudowords, *tacok* and *bitye,* used in the training phase of experiment 2 were used during training.

#### (iii) Procedure

The session consisted of a category-training phase and an EEG category-oddball task phase. The infants were prepared for the EEG recording before the training phase, and were administered the test immediately after completing the training phase.

In the category-training phase, the infant was seated on the carer’s lap on one side of a small stage, facing the experimenter, on the other side of the stage (about 1 m away; figure 2, top right). The experimenter attracted the infant’s attention by calling their name and establishing eye contact, then took one of the objects from a box under the stage and put it on the stage. She ensured that the infant could see the object, labelled it, and offered it to the infant. If the infant did not grasp the object, the experimenter continued manipulating it while communicating about it to the infant. She was talking freely using infant-directed intonation. Each object was labelled at least four times with one of the pseudo words (*tacok* or *bitye*). The presentation of each object was 1 min long. Then, the experimenter put the object back under the table and repeated the procedure with the next object. The six objects were randomly assigned to the two labels, with different combinations of objects for the two labels across infants. The sequential presentation of the six objects respected the following rule: first the experimenter presented three objects naming them with one label, and then she proceeded with the three remaining objects, naming them with the other label.

The EEG category-oddball task phase was identical to the test phase in experiment 2, except that the number of presented trials was not fixed; instead, the sequence was presented as long as the infants were attentive. The behaviour of the infants was video-recorded throughout the session for offline coding. If the infant became fussy, they were given a short break. A rotating spiral with a jingle was presented to reorient their attention to the screen when necessary. The session ended when the infants’ attention could no longer be attracted to the screen.

#### (iv) Electroencephalography recording and analyses

EEG recording parameters, editing and analysis methods were similar to experiments 1 and 2. The infant version of the Hydrocel Geodesic Sensor Nets (Electrical Geodesics Inc., Eugene, OR, USA) included 124 electrodes referenced to the vertex (Cz). EEG processing steps were the same as in experiments 1 and 2 with the exception that we used the frequency range of 7 to 10 Hz for quantifying ERD responses in the infant alpha-band [56]. We also performed a manual rejection of bad epochs, and trials were excluded if infants did not watch the stimuli (judged from video recordings).

We quantified the infant P300 response within the same ROIs as in adults, but in a different time window between 300 and 600 ms (cf. [57]). We quantified the infant-specific Nc component [58], which could indicate orientation towards the infrequent stimulus, as the average amplitude of the waveform is between 300 and 600 ms at the channels around the vertex (see the electronic supplementary material). We measured the alpha-band ERD as the average amplitude in the time window between 300 to 800 ms after the stimulus onset. Infants had to provide 20 artefact-free trials across both conditions. A total of four infants across the two samples had less than 10 accepted trials in the infrequent condition (see the electronic supplementary material for additional analyses excluding these infants).

### (b) Results

#### (i) Experiment 3a

Infants’ responses closely resembled those of adults in experiment 2. The ERPs did not produce differences between conditions, neither over the parietal areas (P300, all *p*s > 0.34) nor over the fronto-central areas (Nc, *p* > 0.14), but an ANOVA on the ERD in the infant alpha range yielded a two-way interaction, category x hemisphere: *F*_1,13_ = 4.68, *p* = 0.050, $\eta_{p}^{2}$ *=* 0.27. We found no other significant effects, all *p*s > 0.20. To resolve the category x hemisphere interaction, we ran separate *t*-tests within each hemisphere to compare the response elicited by the two categories (frequent versus infrequent). We found that infrequent stimuli elicited marginally stronger alpha suppression in the left, *t*_13_ = 1.97, *p* = 0.071, *d* = 0.72, but not in the right hemisphere, *t*_13_ = 0.28, *p* = 0.78, *d* = 0.10. Further *t*-tests comparing the two categories in the left hemisphere revealed that the effect was present in the anterior, *t*_13_ = 2.19, *p* = 0.048, *d* = 0.82, but not in the posterior region, *t*_13_ = 1.40, *p* = 0.19, *d* = 0.41, with 12 out of 14 infants showing the effect, Wilcoxon’s *Z* = −2.42, *p* = 0.016. Thus, the scalp location of the effect matched the site where we found alpha-band desynchronization in adults. Whether the category introduced first or the category introduced second during training was used as the infrequent one had no observable effect on the results: an ANOVA with ‘first trained category’ (frequent trained first versus infrequent trained first) as a between-subject factor and category (frequent versus infrequent) as a within-subject factor revealed no significant effects, all *p*s > 0.229.

#### (ii) Experiment 3b

To confirm these results, we ran a full independent replication of experiment 3a with larger group of 20 nine-month-olds. We used the exact same stimuli and procedure but different experimenters (experiment 3b). As before, an ANOVA on the P300 mean amplitude did not yield any significant results (all *p*s > 0.12), but an ANOVA on the ERD yielded a significant main effect of category, *F*_1,19_ = 5.12, *p* = 0.036, $\eta_{p}^{2}$ *=* 0.21, and a two-way interaction, category x hemisphere: *F*_1,19_ = 14.56, *p* = 0.001, $\eta_{p}^{2}$ *=* 0.43. Separate *t-*tests revealed that infrequent stimuli elicited stronger alpha suppression than the frequent stimuli in the left anterior, *t*_19_ = 3.21, *p* = 0.005, *d* = 0.81, and left posterior, *t*_19_ = 3.09, *p* = 0.006, *d* = 0.85, regions, with 18 out of 20 infants showing the effect, Wilcoxon’s *Z* = −3.47, *p* = 0.001. We also found an interaction of hemisphere x region (see the electronic supplementary material for details). No other main effect and interaction were significant (all *p*s > 0.130). A control ANOVA with ‘first trained category’ as a between-subject factor and category as a within-subject factor only yielded a main effect of category, *F*_1,18_= 5.44, *p* = 0.032, $\eta_{p}^{2}$ *=* 0.23, all other *p*s > 0.160.

We directly compared the alpha-band ERD between the two experiments with the additional between-subjects factor of experiment (3a versus 3b). We found a main effect of category, *F*_1,32_ = 5.84, *p* = 0.022, $\eta_{p}^{2}$ *=* 0.15, and a category x hemisphere interaction, *F*_1,32_ = 16.85, *p *< 0.001, $\eta_{p}^{2}$ *=* 0.35. Separate *t*-tests comparing the two categories in each hemisphere revealed that the effect was significant over the left, *t*_33_ = 4.01, *p* < 0.001, *d* = 0.85, but not over the right hemisphere, *t*_33_ = 0.03, *p* = 0.98, *d* = 0.005. We also found a three-way experiment x hemisphere x region interaction, *F*_1,32_ = 5.95, *p* = 0.02, $\eta_{p}^{2}$ *=* 0.16. Separate independent samples *t*-tests within each ROI showed no effect of experiment (all *p*s > 0.11), suggesting no difference between the two infant samples.

### (c) Discussion

Infants displayed the category oddball effect, with stronger alpha-band desynchronization in response to objects from the infrequent than the frequent category. This pattern closely mirrors the one observed in adults (experiment 2) and indicates that infants successfully categorized the displayed images. Since objects within each category shared no visual features and were shuffled between categories (randomized across participants), category formation must have been triggered by labelling during training. Furthermore, the newly formed categories contained symbolic categorical information, either in the form of category labels or non-linguistic category symbols, as evidenced by the category oddball effect. Such latent non-featural components of category representations were the only commonality between the stimuli. Therefore, labelling appears to induce infants to include both featural and non-featural abstract content into their category representations.

## 5. General discussion

Across three experiments with adults and preverbal infants, we investigated representations underlying label-induced categorization. More specifically, we asked what are the elements of category representations that infants set up when exposed to objects labelled with the same name. First, using a novel EEG category-oddball task in adults (experiments 1–2), we established that alpha-band ERD is a marker of categorization: a stronger alpha-band ERD was triggered by the observation of the stimuli from the infrequent relative to the frequent category, giving rise to a category-oddball effect. Second, we found the same ERD category-oddball effect in nine-month-olds, which provides, to our knowledge, the first direct evidence that infants used labels to group arbitrary, visually unrelated objects into two distinct categories and the category representations they set up contained symbolic elements in addition to featural information extracted from individual category exemplars. These findings corroborate the idea that the link between language acquisition and category formation is bi-directional. When infants have access to spontaneously acquired nonverbal category knowledge, they use it to discover word meanings [29,59]. When they lack such knowledge, as in the present study, they use labels, and possibly other communicative signals [14], as category markers.

Because infants were taught and tested on arbitrary categories whose members did not share any obvious visual features, the categorical responses observed in their EEG could only result from processing summary category markers linked to each of the trained categories. What could these markers be? Human adults deploy both external and internal symbols to represent relevant categories. External symbols are provided through natural language, in the form of words, or via other symbolic systems such as mathematical notation. Internal symbols are believed to be provided via non-linguistic symbolic systems, also known as Languages of Thought [34,60,61], whose psychological reality has recently been experimentally corroborated (for a review see [35]). The current results suggest that by the end of the first year of life, infants may be including symbolic components in their category representations but leave open two further questions: whether these symbols are linguistic or not, and what is the mechanism through which they give rise to the category-oddball effect. Both questions are exciting empirical challenges for future research.

The mechanisms that may be mediating the category-oddball effects could operate on different components of category/object representations. One possibility is that they are implemented through cognitive processes at the level of symbols, involving computations on either linguistic symbols, such as category labels, or non-linguistic category symbols retrieved upon seeing familiar objects. Under this account, the oddball effect would be triggered by the detection of a mismatch between the symbols activated upon exposure to objects from familiar categories. Another possibility is that category-oddball effects result from cognitive processes operating at the level of visual and/or object representations. At least two alternatives should be considered here. First, linking the same linguistic symbol to three different objects may have durably made the representations of these objects closer to each other (e.g. [62]). Second, the associated category symbols may be modulating the visual processing online by augmenting similarity between object representations [19,32,63,64]. Further evidence is needed to disentangle these accounts as well as map the developmental trajectory of the involved mechanisms, as they may change across the lifespan as category knowledge grows significantly, becomes more robust and intimately linked to language. For example, in adults, labels may be more prominent in category representations than labels in infants, who seem to spontaneously retrieve category names only around 18 months of age [65], for an indirect evidence in 12-month-olds, see [66].

To our knowledge this is the first study to provide evidence that infants can form exemplar-based categories made of arbitrary objects that collectively share no obvious visual features. We propose that, upon hearing a new word applied to an object, infants set up a category marker symbol linked to a placeholder concept, in which they collect information about category exemplars. To do so, it is necessary to maintain the category label in the working memory over a short period of time to identify the named objects as exemplars of the same category. It is, however, *not* necessary to encode the label in the long-term memory. This mechanism may not support learning words or mapping them onto object features, but at the very least allows grouping the objects receiving the same label into one category. This can be exploited to generate hypotheses about category membership of novel objects [67,68], such as exploring common visual features. Not finding visual commonalities between all objects receiving the same name, just as in the current task, should not prevent infants from treating them as members of the same category. Although taxing, storing individual category exemplars in a placeholder linked to a category label or its non-linguistic counterpart allows infants to learn about object kinds that are defined by non-obvious (e.g. dispositional) properties, such as functions. Further exploring this phenomenon could significantly advance our understanding of infant categories and conceptual learning. For instance, the mechanism we described may help infants to acquire opaque cultural knowledge [69], which builds on abstract conceptual models in addition to sensory information available via observation. It is also worth noting that although words were the only categorization cue available to participants in experiments 2−3a,b, words are not the only information that people can use during category learning. In fact, we show that in adults the ERD categorization effect, albeit weaker, can be observed without labelling: in the electronic supplementary material, experiment S4a, spatial grouping without labelling whatsoever was enough to trigger categorization. Lastly, it is worth noting that the present effects could in principle result from the following process: rather than mapping the provided label directly onto a category, infants would map it onto the three individual objects. We find this explanation unlikely for two reasons: first, the alpha oddball effect is strikingly similar between adults and infants, suggesting similar cognitive processes between these two age groups. Second, the literature shows that when the same label is applied to different objects infants group them into one category [24], starting as early as 3 to 4 months of age [4].

In our category oddball paradigm, frequency effects in response to visual stimuli could only emerge if the participants treated the individual objects as representing the categories those objects belonged to. We expected to find a frequency effect on the P300 (and/or on the Nc in infants) and alpha-band desynchronization. We observed the P300 modulation only for known real-world categories, characterized by a set of visual features shared among category exemplars (i.e. category-diagnostic features, experiment 1), but not for arbitrary categories composed of objects that did not share any obvious visual features (experiments 2, 3, S4). The alpha-band ERD effects were present across all experiments (but experiment S4b) with different scalp distributions, regardless of whether category tokens shared or did not share visual features. Why alpha-band ERD is more sensitive to category-oddball stimuli compared to the P300 deserves further investigation. Tentatively, we propose that while the ERPs may reflect greater sensitivity to low-level features of the stimuli, the alpha-ERD may be mediated by more abstract differences between the two categories such as category symbols, as discussed above. This proposal is in line with the past evidence that left-lateralized alpha-band ERD has been linked to semantic access [70].

Given that the categories used in experiments 2–3 and S4 were simply arbitrary groupings of strange objects unfamiliar to the participants, it could be speculated that participants formed ad hoc categories [71]. These are novel categories created when familiar ones are not enough to make sense of the environment or achieve one’s goals (e.g. ‘non-touristy places to hang out in Paris’). Although participants lack familiar categories that could be applied to objects in the current experiments, we believe that is unlikely that they resorted to ad hoc categories for the following reasons. These are based on rules conveyed via complex verbal description and as such they are highly unlikely to be available to young infants who do not master natural language. Admittedly, adults could have come up with sets of arbitrary descriptions to interpret the novel object groupings (e.g. ‘the shiny object, the wooden object, and the blue one’), but the fact that neural signatures in the alpha-band ERD domain are similar across experiments 1 (using familiar artefacts) and experiment 2 (using novel objects) speaks against this possibility.

Whether infants categorize objects by retrieving their labels or non-linguistic category symbols representing the trained categories, the implications of our findings are profound. Language is a powerful medium of cultural transmission that can convey the boundaries of concepts and categories used by one’s cultural community [2]. Our findings indicate that labels may play a central role in the identification of novel arbitrary categorical distinctions in human infants, thus allowing them to develop a repertoire of culturally defined artefacts even before fully grasping the underlying concepts. In addition, labelling leads infants to set up representations with abstract feature-independent components, which may be critical for the further development of symbolic cognition.

## Data Availability

The data that support the findings of this study are openly available at the Open Science Framework [72]; the study was not formally preregistered. Supplementary material is available online [73].

## Footnotes

^1^
On 29 August 2014, after our data collection and analyses were finalized, EGI communicated that the anti-aliasing filters of their amplifiers caused a 18 ms delay of EEG recordings with the GES amplifier at 500 Hz sampling rate. This delay was consistent across experiments and conditions and was relatively minor compared to the time windows we used for analyses, not altering our conclusions. In this paper we present the uncorrected recordings.

## Dedication

In memory of Assunta Marozzo and Stanislao Parise.
